# *Nidogen 1 *and *2 *gene promoters are aberrantly methylated in human gastrointestinal cancer

**DOI:** 10.1186/1476-4598-6-17

**Published:** 2007-02-28

**Authors:** Linda Ulazzi, Silvia Sabbioni, Elena Miotto, Angelo Veronese, Angela Angusti, Roberta Gafà, Stefano Manfredini, Fabio Farinati, Takako Sasaki, Giovanni Lanza, Massimo Negrini

**Affiliations:** 1 Dipartimento di Medicina Sperimentale e Diagnostica e Centro Interdipartimentale per la Ricerca sul Cancro, Università di Ferrara, Ferrara, Italy; 2 Dipartimento di Scienze Farmaceutiche, Università di Ferrara, Italy; 3 Dipartimento di Scienze Chirurgiche e Gastroenterologia, Università di Padova, Italy; 4 Max-Planck-Institute for Biochemistry, Martinsried, Germany

## Abstract

**Background:**

Nidogens are highly conserved proteins of basement membranes. Two nidogen proteins, nidogen 1 and nidogen 2, are known in mammals.

**Results:**

We show that CpG islands of both NID1 and NID2 genes are aberrantly methylated in human cancer samples and cancer cell lines. For both genes, methylation was correlated with loss of gene transcription in human cell lines. Furthermore, demethylation of the NID1 and NID2 promoters restored gene transcription, demonstrating that methylation was responsible for silencing nidogen genes. In primary tumors, we detected NID1 promoter methylation in 67% of colon cancer samples and in 90% of gastric cancers. NID2 promoter was methylated in 29% of colon and 95% of gastric cancers. Immuno-staining for nidogen-2 confirmed the correlation between aberrant methylation and loss of nidogen expression also in primary tumors, implying that aberrant methylation was a mechanism for inhibiting nidogens expression in human gastrointestinal tumors.

**Conclusion:**

These results suggest that loss of nidogens expression has a potential pathogenetic role in colon and stomach tumorigenesis. Nidogens are believed to connect laminin and collagen IV networks, hence stabilizing the basement membrane structure. Nidogens are also important for cell adhesion, as they establish contacts with various cellular integrins. Loss of nidogen expression may favor invasion and metastasis of cancer cells by loosening cell interaction with basal membrane and by weakening the strength of the basement membrane itself, first barrier from the connective vascularized matrix.

## Background

Basement membrane is a thin layer of specialized extracellular matrix. Its main components include collagen IV, laminins, heparan sulfate proteoglycan (perlecan) and nidogens. It can be found under sheets of epithelial and endothelial cells and surrounding muscle, fat and muscle cells. It is essential for tissue compartmentalization and maintenance of cell phenotypes; it also supplies stimuli for tissue development and remodeling [1,2].

Among its components, nidogen binds and forms ternary complex with collagen IV and laminin, connecting the two networks and stabilizing the tri-dimensional structure of the basement membrane [3]. Nidogen thus serves the important role of establishing and maintaining basement membrane and tissue architecture.

Nidogen also interacts with cell receptor molecules and controls cell polarization, migration and invasion. Nidogen-1, in particular, interacts with β_1 _family of integrin receptors and with α_v_β_3 _integrin [4-7]. Through interactions with the leukocyte response integrin, nidogen favors neutrophil chemotaxis during the inflammation process. The interactions between cells and basement membranes regulate various cellular processes, including differentiation, proliferation and apoptosis.

In human, two nidogen proteins, nidogen-1 (150 kDa) and nidogen-2 (200 kDa), have been identified. The two proteins share a 46% primary sequence identity and a very similar three dimensional structure, consisting of three globular domains connected by a flexible link and a rod [8,9].

Both nidogens are co-expressed in various tissues and interact with collagens I and IV and perlecan at comparable level. Differently, *in vitro *binding of nidogen-2 to laminin γ1 is weaker than for nidogen-1. Also, nidogen-2, differently from nidogen-1, does not interact with fibulins. Both nidogens are also cell-adhesive. The above features indicate that the two proteins might fulfill similar if not identical functions, may be interchangeable in many, but not all the cases and may compensate each other deficiency [10,11]. This suggestion is confirmed by the findings that both nidogen-1 and nidogen-2 are ubiquitous components of basement membranes underneath epithelia of most of the major organ systems [10]. Furthermore, while deficient mice for either nidogen-1 or nidogen-2 [12,13] have no overt phenotypic abnormalities and basement membranes appear to be normal, preliminary reports of double mutants indicate a perinatal lethal phenotype and altered basement membrane ultrastructure [12]. At least in the case of nidogen-1 knock-out, nidogen 2 compensation was demonstrated. An increase in nidogen-2 expression and unexpected high affinity of nidogen-2 for laminin γ1 was discovered, thus explaining the lack of phenotype of the NID1-null mice [11].

In spite of the fact that it is established that abnormal stromal microenvironment contributes to tumor formation and progression and nidogens play a key role in the maintenance of the structural integrity of basement membranes, which establish a barrier for cell movement, migration and invasion, nidogen defects have not been linked to human cancer. Here, we report that methylation of nidogen 1 and 2 promoters is responsible for loss of their gene expression and is frequent in human gastrointestinal tumors.

## Materials and methods

### Cell lines

The cell lines used in this study were MCF7, MDA-MB-231 and BT-20 from breast carcinoma, SW48 and RKO from colon carcinoma, HEP3B from hepatocellular carcinoma, RT4 from bladder carcinoma, SiHa and HeLa from cervical carcinoma, SK-LU-1 from lung carcinoma, HEY from ovary carcinoma, G401 from a renal rhabdoid tumor, U937 from a histiocytic lymphoma, K562 from a blast crisis of a chronic myeloid leukemia, G361 from a melanoma, RD from a rhabdomyosarcoma. These cell lines were obtained from the American Type Culture Collection (ATCC) and grown in Iscove Medium (Sigma-Alldrich) supplemented with 10% Fetal Bovine Serum (Sigma-Alldrich) at 37°C in 5% CO_2 _atmosphere.

### Treatment with zebularine and trichostatin

Treatment of cells with Zebularine (synthesized at the Department of Pharmaceutical Sciences, University of Ferrara) was carried out for 72 hours at 500 μM concentration. Trichostatin (TSA) (Sigma-Alldrich) treatment was for 24 hours at 500 nM. When combined with zebularine, TSA treatment was added during the last 24 hours. Immediately after treatments, cells were harvested for DNA and RNA purification.

### Tumor and normal primary samples

Samples from primary tumors and non-neoplastic specimens were from colorectal, gastric tissues, and from peripheral blood leukocytes. Unselected gastric carcinomas were collected from 20 patients. Colorectal carcinomas (CRC) were collected from 49 patients. Additional files 1 and 2 describe the characteristics of tumor samples. Normal controls included biopsies of stomach mucosa from 13 patients with gastric ulcers and colon mucosa from 24 patients with diverticulosis. All patients included in the study had a histological confirmed diagnosis. Informed consent was obtained from each subject included in the study.

### DNA purification and methylation specific PCR

**Figure 1:**
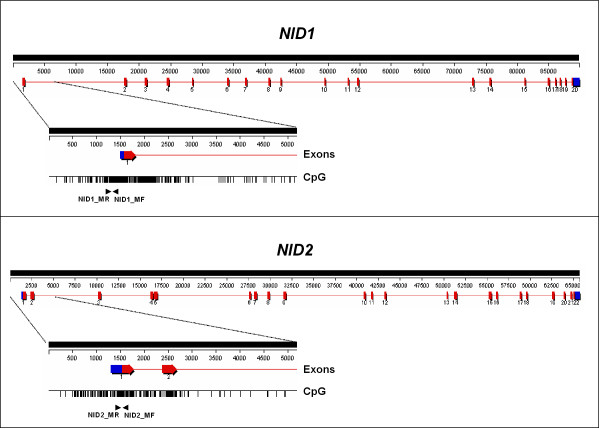
Schematic diagrams of the human NID1 and NID2 genes and their 5' CpG islands. Size of the genes and exon composition are shown in the upper parts of the schemes. In the lower parts, the 5' of the genes is enlarged and the presence of a dense region of CpG dinucleotides (a CpG island) is displayed. The CpG islands overlap with the first exons of the genes. **Exons**, arrowed boxes separated by thin lines; blue parts are non-coding regions, red parts are coding regions. **CpG**, each vertical bar represents the presence of a CpG dinucleotide. Arrows indicate the location of primers for MSP amplification (NID1 amplified region: from nt 986318 to nt 986461 of sequence NT_004836 version 17; NID2 amplified region: from nt 33535309 to nt 33535449 of sequence NT_026437, versione 11). All primers were designed on predicted bisulfite-modified nucleotide sequence (see Supplemental Figures 1 and 2).

Genomic DNA was isolated from cell lines and tissue by standard treatment with sodium dodecyl sulfate and EDTA in the presence of 200 μg/ml of proteinase K, followed by phenol/chloroform extraction and ethanol precipitation. DNA from peripheral blood was isolated using the Wizard genomic DNA purification kit (Promega) in accordance with manufacturer's instructions. The methylation status of gene promoters was determined by Methylation Specific PCR (MSP) as described by Herman et al [14]. The oligonucleotides for amplification of NID1 and NID2 promoter regions are described in Table 1, their location in the CpG island of the two genes is illustrated in Figure 1 and detailed in Additional files 3 and 4. PCR reactions were performed with the HotStar Taq polymerase (Qiagen) in a total volume of 12 μl with the following condition: 40 cycles of denaturation at 95°C for 30 sec, annealing at temperatures indicated in Table 1 for 30 sec and extension at 72°C for 1 min. For bisulfite sequencing of NID1 promoter, after amplification with primers NID1-UM-BFw2/NID1-UM-BRv1 (Table 1), PCR products were sequenced using primers NID1-UM-BFw2 or NID1-UM-BRv1 using an automated sequencer ABI377.

**Table 1:** NID1 and NID2 oligonucleotides for MSP and RT-PCR analyses

Gene	Method	Primer 1	Oligonucleotide sequence^a^	Primer 2	Oligonucleotide sequence	Product size	T (°C) annealing
NID-1	MSP methyl	NID1_MF	GAGGGTTTCGTTTCGTTTAGC	NID1_MR	AACGCCGTTCGCTAAAAATCG	144	58°C
NID-2	MSP methyl	NID2_MF	TAGTATTGGTAACGACGATAGTATC	NID2_MR	AAATTCGAAACTAACGCGACACG	141	58°C
NID-1	MSP unmethyl	NID1_UF	GAGGGTTTTGTTTTGTTTAGT	NID1_UR	AAAAACACCATTCACTAAAAATCA	147	51°C
NID-2	MSP unmethyl	NID2_UF	TAGTATTGGTAATGATGATAGTATT	NID2_UR	AAATTCAAAACTAACACAACACA	141	51°C
NID-1	RT-PCR	NID1_181F	AGGAGCTCTTTCCCTTCGGC	NID1_354R	CGGGGGTTCACTCGTAGCAA	174	60°C
NID-2	RT-PCR	NID2_722F	TAGGCGCTTACGAGGAGGTCAA	NID2_813R	TATCAGACCCATCAGATGCCAAAAC	92	63°C

^a ^All primers for MSP were designed on predicted bisulfite-modified DNA sequences (see Additional files 3 and 4)

### RNA purification and RT-PCR

Total RNA was prepared using the TRIzol Reagent (Invitrogen Life Technologies, Carlsbad, CA) in accordance to manufacturer's instructions. RNA was reverse transcribed using Superscript II Reverse Transcriptase (Invitrogen, Carlsbad, CA) in the presence of oligo-dT and random primers. The primers used for NID1 and NID2 cDNA amplification are shown in Table 1. Detection of the GAPDH gene expression was used for cDNA quality control and normalization. Oligonucleotides for GAPDH cDNA amplification were: GAPDH-RNA1107F (5'-CTA TAA ATT GAG CCC GCA GCC-3') and GAPDH-RNA1257R (5'-CCC AAT ACG ACC AAA TCC GT-3'). All PCR reactions were performed with Hot Star Taq polymerase (Qiagen) in a total volume of 12 μl, with the following condition: 35 cycles (25 for GAPDH) of denaturation at 95°C for 30 sec, annealing at 60°C for 30 sec and extension at 72°C for 30 sec. Nucleotide sequence of PCR product was determined to confirm specificity of RT-PCR products.

### Nidogen immunohistochemical analysis

Immunohistochemical analysis of nidogen-2 expression was performed on formalin-fixed paraffin-embedded tumor tissue sections using the ultraView Universal DAB detection system and the BenchMark XT automated immunostainer (Ventana Medical Systems, Tucson, AZ, USA). Rabbitt antisera against nidogen-2 was produced by one of the co-author (TS).

## Results

### Human cancer cell lines are methylated at the NID1 and NID2 promoters

We detected *NID1 *as a gene reactivated by treatment with the demethylating agent zebularine in the human cancer cell line MDA-MB-231 (data not shown). To confirm that reactivation was induced by promoter demethylation, we analyzed the methylation status of the *NID1 *promoter in the MDA-MB-231 cell line and in several other human cell lines. In addition, since two nidogen homologous genes exist in the human genome, we also analyzed the methylation status of the promoter of the *NID2 *gene.

**Figure 2:**
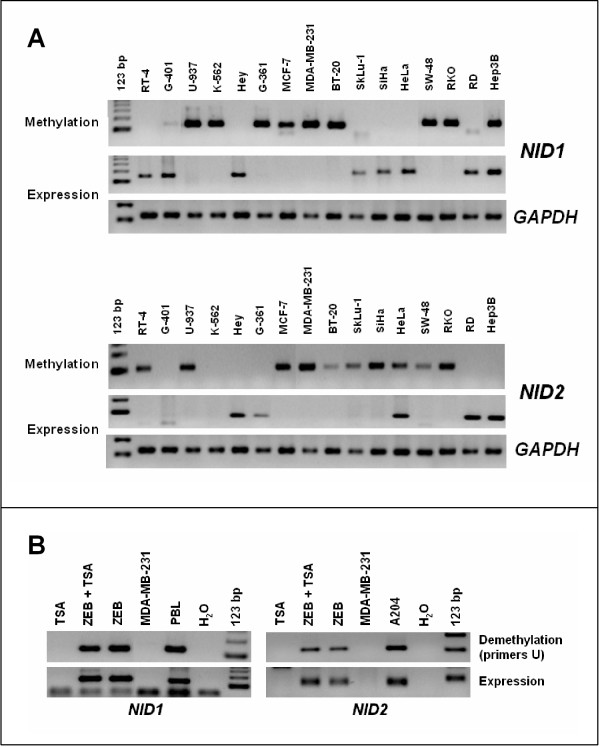
(**A**) NID1 and NID2 promoter methylation and gene expression in human cancer cell lines. With few exceptions, there is a clear correlation between gene expression and lack of promoter methylation. Each sample was checked and confirmed for quality and equal cDNA amount used by analyzing expression of GAPDH by RT PCR amplification (GAPDH experiment was identical for both genes). (**B**) NID1 and NID2 genes are reactivated by the DNA demethylating agent zebularine. The panels show the effect of zebularine (ZEB) and trichostatin (TSA) on DNA demethylation and gene expression in the human cell line MDA-MB-231. Zebularine, alone or in combination with TSA, induced expression of both genes, which was absent in the parental untreated cell line and in the treatment with TSA alone. PBL, peripheral blood leukocytes, and the cell line A204 were used as positive controls. 123 bp, Molecular weight marker 123 bp ladder.

Both promoter regions of *NID1 *and *NID2 *genes include a dense CpG island spanning about 2 kb around the first exon (Figure 1). To determine whether aberrant methylation occurs at this region, we analyzed 16 human tumor cell lines by methylation specific PCR (MSP) using the primer sets MF/MR (Table 1 and Additional files 3 and 4). 56% (12/21) of the cell lines revealed methylation at the NID1 promoter and 62% at the NID2 promoter (Figure 2A). Nucleotide sequence of PCR products confirmed the methylation of all the cytosines within CpG dinucleotides. To determine whether methylation was associated with loss of gene expression, we analyzed the expression of the NID1 and NID2 genes by RT-PCR in the same 16 cell lines. We confirmed that the presence of promoter methylation was associated with loss of gene expression with only two exceptions: NID1 in the Hep3B cells and NID2 in the HeLa cells. Furthermore, with the exception of G401 and K562 for the NID2 gene, both genes were expressed in the unmethylated cell lines, implicating a direct relationship between promoter methylation and loss of gene transcription. To confirm the role of DNA methylation in gene transcriptional repression, we treated MDA-MB-231 cells, where NID1 and NID2 are both methylated, with the demethylating agent zebularine alone or in combination with TSA. Indeed, demethylation of the NID1 and NID2 promoters restored gene transcription, demonstrating that promoter methylation was responsible for the silencing of nidogen genes (Figure 2B).

### NID1 and NID2 promoter methylation in human primary gastrointestinal neoplasms

**Figure 3:**
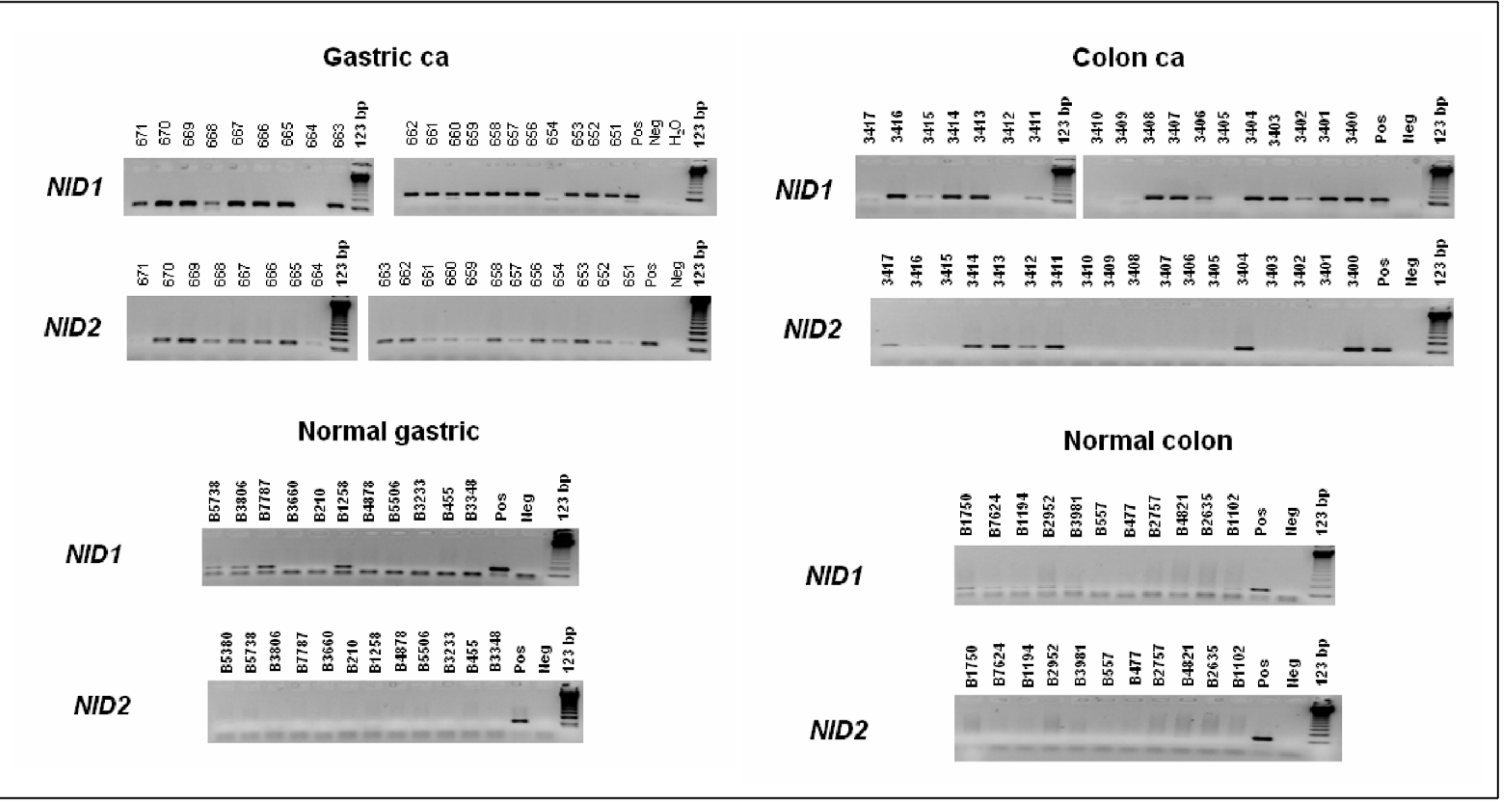
Methylation at the NID1 and NID2 gene promoters in human colon and gastric cancer. DNA methylation was detected by methylation-specific PCR. DNAs were modified by bisulfite treatment. PCR was performed using primers specifically designed to amplify the methylated forms (see **Table 1**). The frequency of aberrant methylation in normal and cancer samples is shown in **Table 2**. Molecular weight marker (123 bp), Controls were as indicated in Figure 2.

We investigated the presence of aberrant methylation at the NID1 and NID2 promoters in primary gastrointestinal neoplasms. Data are summarized in Table 2 and representative results are shown in Figure 3. By using MSP with the MF/MR primer set (Table 1), we analyzed 49 colorectal and 20 gastric carcinomas, as well as 13 gastric and 24 colonic mucosas from non-neoplastic specimens. For the NID1 gene, we detected promoter methylation in 67% (33/49) of the colon and 90% (18/20) of the gastric carcinoma samples. Methylation of this gene promoter was also detected, at lower frequency and signal intensity, in colon and gastric mucosas of non-neoplastic specimens. Nucleotide sequencing of PCR products detected in normal tissues revealed only sporadic methylation at few CpGs in the context of essentially non-methylated CpG islands, while methylation was detected at all CpGs in tumor samples (data not shown). For the NID2 gene, we detected promoter methylation in 29% (14/48) of the colon and 95% (19/20) of the gastric carcinoma samples. No methylation of the NID2 gene promoter was detected in colon and gastric mucosas from non-neoplastic specimens.

**Table 2:** NID1 and NID2 promoters DNA methylation

	***NID1 *methylation (%)**	***NID2 *methylation (%)**
Stomach, carcinoma	18/20 (90)	19/20 (95)
Stomach, normal mucosa	5/13 (38)*	0/13 (0)
Colon, carcinoma	33/48 (69)	14/48 (29)
Colon, normal mucosa	8/24 (33)*	0/24 (0)

* Generally, 10–100 fold weaker signals than in tumor samples. These samples displayed only few CpG dinucleotides methylated in an overall non-methylated CpG island

**Figure 4:**
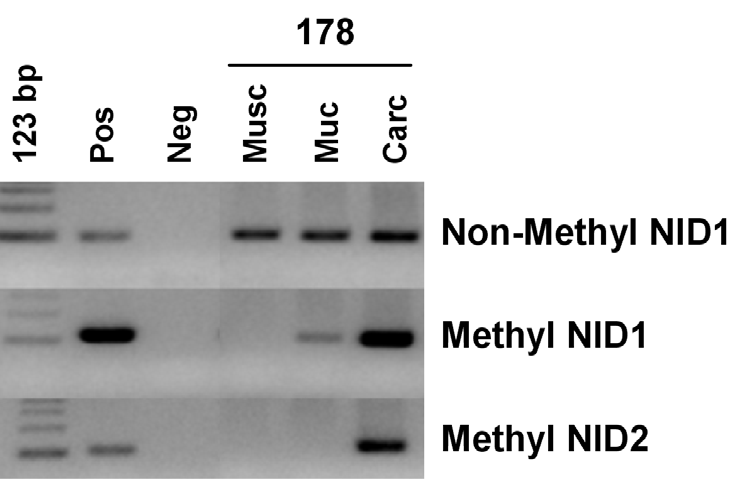
Methylation specific PCR for NID1 and NID2 promoter in DNAs from tissue dissected cancer (Carc), mucosa (Muc) and *muscolaris *(Musc) from the same patient, showing strongly positive signals only in carcinoma samples and not in mucosa and *muscolaris *(except a weak signal for NID1 in mucosa). Molecular weight marker (123 bp), Controls were as indicated in Figure 2.

**Figure 5:**
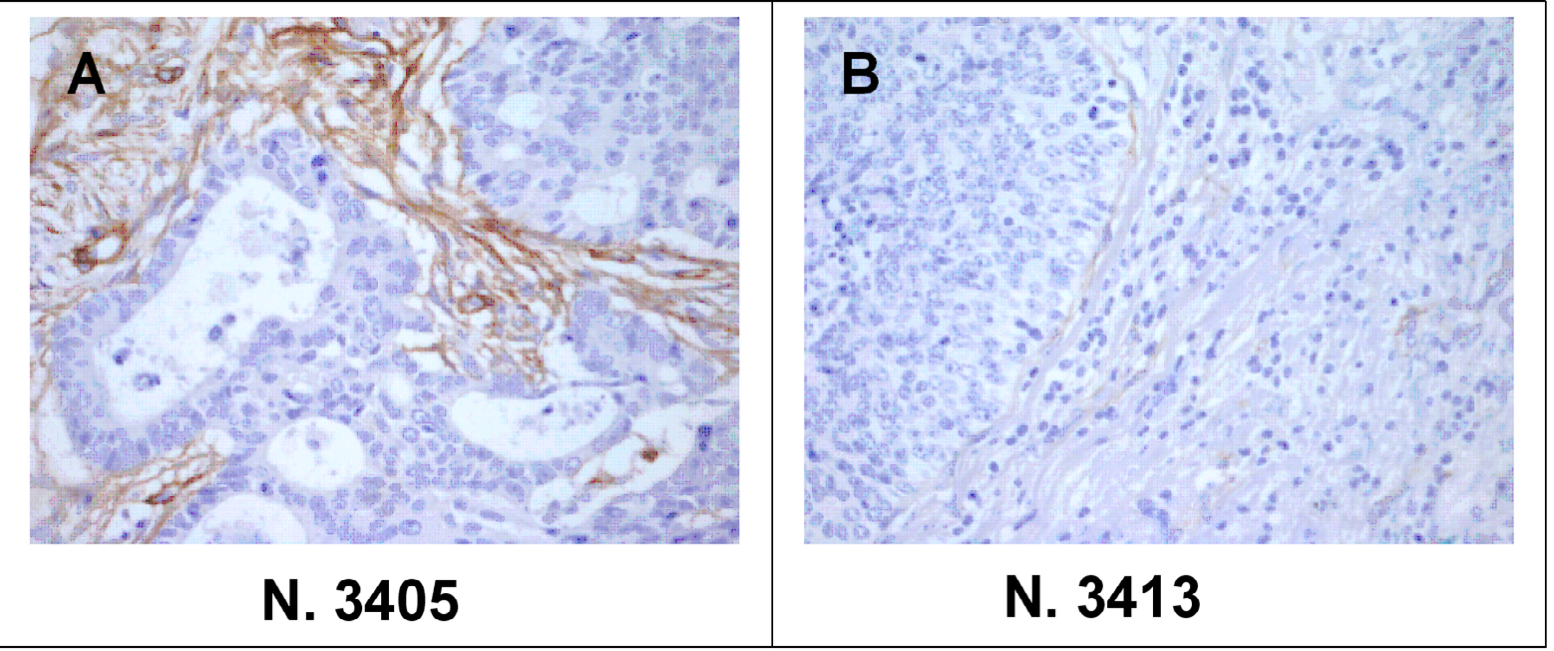
Immunohistochemistry analysis of nidogen-2 expression in human colon carcinomas. (A) Sample 3405, a moderately differentiated adenocarcinoma that does not present aberrant methylation of the NID2 gene promoter, shows a strong immunoreactivity for nidogen-2 in the stroma. (B) Sample 3413, a poorly differentiated adenocarcinoma that presents aberrant methylation of the NID2 gene promoter, shows a nearly complete lack of immunoreactivity for nidogen-2. (Original magnification, 200×).

To further confirm that aberrant nidogen methylation was associated with cancer tissues, we analyzed DNAs derived from dissected cancer, mucosa and *muscolaris *from the same patient. These experiments confirmed that aberrant methylation was confined to cancer tissue (Figure 4). Furthermore, analysis of nidogen-2 by immunostaining in primary tumors confirmed that only tumor samples presenting aberrant methylation lacked nidogen expression (Figure 5).

## Discussion

Removal of basement membrane is essential for cell invasion [15], a regulated process that occurs in trophoblast implantation, organogenesis and angiogenesis [16]. In tumorigenesis, invasion through the basement membrane is a feature of malignancy [17]. Disruption of the integrity of the basement membrane creates an invasion-permissive environment, which may promote cancer cell proliferation and invasion [18]. Basement membrane abnormalities may cause an increase in tumor susceptibility [19] by bypassing the need for degradation of the basement membrane and by triggering pro-proliferative effects on tumor cells via interaction with stromal fibroblast growth factors, cytokines, and/or matrix proteases [20].

Examples of abnormalities of basement membranes in human cancer have been described. Malignant melanomas lacked basement membranes when they migrated into the dermis, a process accompanied by a drastically reduced staining for collagen type IV and nidogen [21]. Wilms' tumors are coated with a layer of amorphous material that does not represent a basement membrane, since it contains polysialic acid but not laminin, laminin-nidogen complex, or low density proteoglycan [22]. The role of basement membrane in normalization of tumor cells was functionally proven, for example, by experiments with pre-malignant breast epithelial cells, which undergo growth arrest and differentiate in the presence of a reconstituted physiological basement membrane [23]. Overall, these observations suggest that major alterations in the composition of basement membranes are important in tumor establishment or progression.

Here, we report for the first time that loss of nidogen expression, a common basement membrane protein, is frequent in human gastrointestinal tumors. The mechanism underlying nidogen loss is aberrant promoter methylation of both NID1 and NID2 genes. Our results indicate that aberrant methylation at nidogen genes is an epigenetic defect that is absent in non-neoplastic mucosa and *muscolaris*, and potentially imparts a selective advantage to tumor cells. The lack of expression of nidogen proteins, which are both present in the basement membranes of the normal adult intestine [10], impairs the possibility of compensation for constitution of basement membrane. It has also been reported that nidogen can protect laminin-1 against proteolysis [1], suggesting that nidogen absence may cause additional basement membrane proteins degradation and remodeling. Nidogen loss will certainly affect basement membrane structural integrity. Potentially, this mechanism may facilitate the route to invasion for genetically altered epithelial cells and favor metastasis by promoting angiogenesis.

## Supplementary Material

Additional file 1 Clinical and pathologic features of the 49 colon cancers included in the study. Table describing clinical and pathologic features of the colon cancers included in the study Click here for file

Additional file 2 Clinical and pathologic features of the 20 gastric cancers included in the study. Table describing clinical and pathologic features of the gastric cancers included in the study Click here for file

Additional file 3 Predicted bisulfite-modified nucleotide sequence of the top strand methylated NID1 CpG island. Figure presenting the nucleotide sequence on which the methylation specific PCR primers have been designed for the detection of methylation in the NID1 CpG island. Click here for file

Additional file 4 Predicted bisulfite-modified nucleotide sequence of the top strand methylated NID2 CpG island. Figure presenting the nucleotide sequence on which the methylation specific PCR primers have been designed for the detection of methylation in the NID2 CpG island. Click here for file
