# Supplementary material for: Nidogen 1 and 2 gene promoters are aberrantly methylated in human gastrointestinal cancer
Source: Mol Cancer. 2007 Feb 28;6:17. doi: 10.1186/1476-4598-6-17 (PMC1831485; doi:10.1186/1476-4598-6-17)
Supplement: Additional file 1 — Clinical and pathologic features of the 49 colon cancers included in the study. Table describing clinical and pathologic features of the colon cancers included in the study [file 1476-4598-6-17-S1.doc]

**Additional file 1.** Clinical and pathologic features of the 49 colon cancers included in the study

| **Sex** |  |
| --- | --- |
| Male | 17 |
| Female | 32 |
| **Age (years)** |  |
| < 60 | 17 |
| 60-70 | 18 |
| > 70 | 14 |
| **Tumor site** |  |
| Right and transverse colon | 23 |
| Descending and sigmoid colon | 19 |
| Rectum | 7 |
| **Tumor stage (TNM)** |  |
| I | 5 |
| II | 22 |
| III | 19 |
| IV | 3 |
| **Tumor type (WHO)** |  |
| Adenocarcinoma | 40 |
| Mucinous adenocarcinoma | 7 |
| Signet-ring cell carcinoma | 2 |
| **Grade of differentiation (WHO)** |  |
| Well/moderate | 35 |
| Poor | 14 |
