# Supplementary material for: Nidogen 1 and 2 gene promoters are aberrantly methylated in human gastrointestinal cancer
Source: Mol Cancer. 2007 Feb 28;6:17. doi: 10.1186/1476-4598-6-17 (PMC1831485; doi:10.1186/1476-4598-6-17)
Supplement: Additional file 2 — Clinical and pathologic features of the 20 gastric cancers included in the study. Table describing clinical and pathologic features of the gastric cancers included in the study [file 1476-4598-6-17-S2.doc]

**Additional file 2.** Clinical and pathologic features of the 20 gastric cancers included in the study

| **Sex** |  |
| --- | --- |
| Male | 13 |
| Female | 7 |
| **Age (years)** |  |
| < 60 | 8 |
| 60-70 | 6 |
| > 70 | 6 |
| **Tumor site** |  |
| Cardia | 4 |
| Fundus | 2 |
| Body/antrum | 14 |
| **Level of infiltration (TNM)** |  |
| pT1 | 1 |
| pT2 | 7 |
| pT3 | 10 |
| pT4 | 2 |
| **Lymph node involvement** |  |
| Absent | 7 |
| Present | 13 |
| **Laurén classification** |  |
| Intestinal | 13 |
| Diffuse | 7 |
| **Grade of differentiation (WHO)** |  |
| Well/moderate | 11 |
| Poor | 9 |
