# Supplementary material for: Nidogen 1 and 2 gene promoters are aberrantly methylated in human gastrointestinal cancer
Source: Mol Cancer. 2007 Feb 28;6:17. doi: 10.1186/1476-4598-6-17 (PMC1831485; doi:10.1186/1476-4598-6-17)
Supplement: Additional file 3 — Predicted bisulfite-modified nucleotide sequence of the top strand methylated NID1 CpG island. Figure presenting the nucleotide sequence on which the methylation specific PCR primers have been designed for the detection of methylation in the NID1 CpG island. [file 1476-4598-6-17-S3.doc]

| 1 attcgtttta ggtaaagttt ataagtttta cgtgagtgtt gtttaaggcg tagtgttttt  61 aggttagatt gggagtatag ttattttacg ggtaggatag gttgtagtcg ttgcgtttgt  121 agttttaatt tttttttttt ggttaatttt ttatattttt ggaatgtata taagttttat  181 atgaaattta tatggtgggt ggtcgaggaa ggtagcggta cgaaaggatt ttaataagtt  241 gatagcgatt ttaggttcgg ttagttacgg ggaggattta gggagttgta ggttatttgg  301 tttcgttttt cgtttggggt tttcgtggtg ataatgtttg gttataggaa atgggattat  361 tgggagtttt gtgtttttcg ttttggattt tggattttga aacgagttta attggaaagt  421 agtattattg ttaagatata ttcggagttt atatataaat ataggaggtt gtcggcgaaa  481 ttttttcgta gttataagag tttcggggtt cgcgcgtaga ttgcgattta gtttcggttt  541 ttgttttgcg gaggtttcgt gcgagaattt ggttttattt gggatcggtt aggttttagt  601 ttcgaaggga ttggatagtt gagggacgcg gattgatttt tttgcggcga tgtagtttat  661 agtcgtcggg gaggaagttt cgcggggttt atgggtgtcg gggttacggt tcggggcgcg  721 cggagttatt tagatttcgt gattcgtttt tcggatagta gggatcgggt cgggaagatt  781 ttcgcggatt ttgtagaggt gggagatttt gcgtcgtgtt cggtgtttcg gcggttagcg  841 ggttcgggtt ttcgtttgtt acgtttaagt tcgttcggtt ttacgggcgt tttcggttcg  901 tcgcgttttg tagtttttcg ttcgtttttt cggggtttat ttacgtagat tgcgtcgatg  961 tcggatttgt cgtagaagcg gagcgtttta tttagtttta gggtaggaga gacgaagtta  1021 ttttcgtttt ttagttttag gtttttttgt tcggggtcga agggaaagag tttttggcgg  1081 tttaggtagt ttataggttt cgttagtagt agcggtagta gtagcgttcg cgtttacgta  1141 gttcggattt ggttgttcga ggttaatatg ttttcgaatt gcggtttcgt aaattcggtt  1201 tcgcggttag gatagaggaa ggcggggatg taatcggacg tttttggtag tttttttttt  1261 tttcgttttt cggcgttttt atttcgggaa ataggggagg ggagcggagg ggagagcg**ga**  NID1-MF  1321 **gggtttcgtt** **tcgtttagc**g ggcgcgtcgt ggggtagttt aatggtcgcg agggggaggg  1381 cgaggggcga agtcgaattt ttcgttttgg gcgtcggtgt ttgcgatttt cgaaattagt  NID1-MR  1441 **tcgattttta** **gcgaacggcg tt**ttcgggag ggggagtaga gcgcgggatt cgtatcgtgg  1501 ttttttgatt agggttgggt cgtggagagt cggcgacgtg gaaacgtttt ggatttagga  1561 acggcgttta ttttattgtt gttcggtcgt gttcggggtt taattcgttt gattttggta  1621 ggaagacggg gtgggagttg ggaattagtt aagggataga gttattaata cgtttatggg  1681 tttatttatt tatttattgt gcgttttttg agtgtttgat tttgtgtttt ttattgtgga  1741 ggttcgttag gaaacggggt tttatttttt gaggtaaagt tattagtgta taatataagt  1801 tagtgttaga tgggggtttt aagttattaa agatattagg gttggaaaaa ttggcgggga |
| --- |
| **Additional file 3**. Predicted bisulfite-modified nucleotide sequence of the top strand methylated NID1 CpG island. Primers of Table 1 are indicated by bold underlined sequences. |
