# Supplementary material for: Nidogen 1 and 2 gene promoters are aberrantly methylated in human gastrointestinal cancer
Source: Mol Cancer. 2007 Feb 28;6:17. doi: 10.1186/1476-4598-6-17 (PMC1831485; doi:10.1186/1476-4598-6-17)
Supplement: Additional file 4 — Predicted bisulfite-modified nucleotide sequence of the top strand methylated NID2 CpG island. Figure presenting the nucleotide sequence on which the methylation specific PCR primers have been designed for the detection of methylation in the NID2 CpG island. [file 1476-4598-6-17-S4.doc]

| 1 aagtattttt ttttttaagg gagtgattat ttgttagggt tttagaagat gtttagagat  61 aacgtttttt tttattttta agttatttta gttttgttgt ttttaaggtt aggtttagcg  121 taagagtagg tttagaaata atttcgagtt agtttttggg tcgttgtttt agaatttaag  181 tttatttttt aattttttta gtagttaaat atttttggtt atttattttt ttcgagggta  241 gcgtttcgcg tttgattttt tcgtaagcgt ttatttgttt ttaggtggtt aggaaggcgt  301 gggtgggggt aaagcgcgta gagcgcggga agttagcgcg tatatagcgg gcggttaggt  361 ttagtattgt gggggaggtg ttttttcggt ataggattcg gtttttgtcg tggttcgtgt  421 cgatgttcgt tagaaaaggg gcgatggtcg ggaagtcggt ggggaaatta tagtttatat  481 attgcgtttt tttggggaag ttttgagtgg agatgatgtt gttggtgttt atttgggaga  541 ggagagggat aaaaaggtga tagtcgttta agtttaagga cgttattttt tcgcgtaggg  601 agggaaggga attgatttag gtaaaaagtt tttttttttt tttttattgg ttagggagtt  661 tggatttagg gaaataagcg taatttttgt gattgtttta gtttcgcgtt gtagtaggtt  721 taggggtttt aaaattcgtt tatttatttt tttatttttt ttataatttt ggagttttat  781 gttttcggag aggaagtttt taggtttgaa gagggaatat tttagtttcg tagacgggta  841 tagttttttt gcggttttaa ttgtaaagtt cgttggtcgt tttagtaacg ttattttttt  901 tgtttgggat gcgtacgtgg ttgcgaataa tttttgtttt tttttttagg ttatttttgt  961 ttttttatag tagcggggta ggtagcggta tcgttttttg gtcgggtatt atcgtaaggg  1021 tgttgggggg ttttaggagt ggggtttgtt tttttttttt tggaggtagg gttttcgtga  1081 gatcgatttt agggaagaag ttgcgggaaa agtgttagga gggggttgaa tttacgtaga  1141 ggttgttgaa tcgggtttcg tagaagtgta ggggattcgt tagttttatt acggttgagt  1201 tttcgtcgtt gtttttttgt aggagttggt ttttttacga tttttcgtgt gggaagagtt  NID2-MF  1261 cgtttgggtg tagcgtcgcg gttcgtaata ttagtaacgg tagtagtag**t** **agtattggta**  1321 **acgacgatag** **tatc**ggtcgt tcggttattt ggtttttttt tatgttcgtt cggtcgtgcg  1381 tttattcgtt gtataacgcg tttcgtttcg gtttttagtt tattttt**cgt** **gtcgcgttag**  NID2-MR  1441 **tttcgaattt** ggattttcgt gggcgtttgg gcggggcggg attttaggtt tatttagtaa  1501 gttaattggt tgaggttttt ggttggggat taattatcgg ttttgagaaa gttcggtagt  1561 ggttattata tttggttttt gttaattttt ttaaggtagt ggtcgttgga gtagcggggt  1621 tggcggggta aaagtttttg gttagggttg tttggagttg ttttttttat ttcgttttta  1681 gggagttttt gggttatttt tttattcggg ttgtttcgcg gtttttaagg agttttattt  1741 tcgggattaa atggttcgta aggtttgggg tagcggcgtt gtaggagatg agtttagcgt  1801 aaagggaatt tcgtagcggt gagtgcggtt gttggtttgt gcgttgtggt tttaataggt  1861 tggtagggcg cgggcgggtg gcggggttgt ggtatgagtt ttgttttttg ttttggggtt  1921 tcgggcgttt ttggttttgg aggtttggtt atcggttcga aaagtaaagg gatattgtcg  1981 tggttttttt ggtcgtttcg ttttatttag agacgttttg aggagaagcg agtttttgta  2041 cgaaggtaga gggttgattt ttaagttttg tttttttttt gaaggtcggt cgttagggaa  2101 gatttggtta ttttttggaa gtattttttt tcgggaaaat cgtagtagga aagattatga  2161 aatattattg tgtgtgattt gtttcgtttg gggagtagga tgaggtattt cgtttggtta  2221 ttaggtagaa aggggcgagt cgcggggatg ggaagatttt gattttttgt ttattgttgt |
| --- |
| **Additional file 4**. Predicted bisulfite-modified nucleotide sequence of the top strand methylated NID2 CpG island. Primers of Table 1 are indicated by bold underlined sequences. |
